# Functional differentiation in operation: latent violence and colonial labor governance

**DOI:** 10.3389/fsoc.2026.1898394

**Published:** 2026-08-27

**Authors:** Kosuke Sakai

**Affiliations:** 1 Graduate School of Human Development and Environment, Kobe University, Kobe, Japan; 2 Faculty of Sociology, Bielefeld University, Bielefeld, Germany

**Keywords:** colonial governance, functional differentiation, German South West Africa, historical sociology, labor governance, Niklas Luhmann, physical violence, systems theory

## Abstract

**Background:**

This article develops a Luhmannian historical-sociological approach to functional differentiation by examining colonial labor governance in German South West Africa after the Herero and Nama War. It asks how the possibility of physical coercion was reconnected to everyday governance through labor regulation, police supervision, and vocabularies of protection and proper treatment.

**Methods:**

The article uses a historical-sociological and conceptual method. It reconstructs Luhmann's theory of power, functional differentiation, and the potentialization of violence, and relates this theoretical perspective to a historical analysis of colonial labor governance. The analysis draws on colonial ordinances, contemporary administrative debates, and secondary historical scholarship on labor, policing, violence, and population governance.

**Results:**

The analysis shows that physical coercion made manifest through war and camps was reconnected to everyday governance through a regime of registration and labor control. This regime made African workers observable as laboring subjects whose mobility, employment, treatment, and punishability could be regulated. Legal, contractual, protective, and police vocabularies became relevant through the problems they made visible and the forms of processing they enabled.

**Conclusion:**

The article argues that functional differentiation can be approached as an operationally grounded historical sociology. Rather than treating functional differentiation as a completed structure of modern society, it examines how concrete operations of communication made problems observable as matters of law, labor, policing, protection, and education. The case of German South West Africa offers a historical-sociological point of departure for rethinking the potentialization of violence within the global formation of modernity.

## Introduction

This article takes up Niklas Luhmann's theory of functional differentiation as a mode of historical-sociological observation. Rather than treating functional differentiation as a completed structure of modern society, it asks how different forms of observation operate and become connected in concrete historical settings. In doing so, it extends Luhmann's historical sociology to the case of colonial labor governance in German South West Africa.

After the Herero and Nama War, colonial rule in German South West Africa reorganized African life around the problem of labor. Survivors of war and camps were registered, supervised, and tied to employment relations through legal and police procedures. At the same time, the treatment of workers became a problem of governance: employers, police officers, and colonial administrators discussed punishment, desertion, food provision, illness, and protection as conditions for maintaining labor power.

The article focuses on this connection between labor governance and the possibility of physical coercion. In Luhmann's theory of power, physical violence is potentialized as a background condition of political communication. In the colonial setting, however, violence made manifest through war, camps, and coercive labor was reconnected to everyday governance through legal classification, police supervision, and vocabularies of protection and proper treatment. By tracing this process, the article explores how a Luhmannian perspective on functional differentiation can be developed as an operationally grounded form of historical-sociological analysis.

## Observing functional differentiation in operation

How can Luhmann's systems theory be taken up as a resource for observing modern society? This question requires returning to the historical-sociological character of his theory. Luhmann has often been read as a general theorist of modern society. At the same time, however, he was also a historical sociologist who analyzed the correlation between social structure and semantics. This historical-sociological project was developed in critical engagement with several earlier approaches to knowledge, concepts, and culture (cf. Sakai, 2021, 31ff.).

Luhmann distanced himself from Mannheim's tendency to relate knowledge to the social location of particular classes or groups, while retaining the sociology-of-knowledge question of how forms of knowledge are socially conditioned. He also drew on Koselleck's *Begriffsgeschichte*, especially its concern with the relation between conceptual change and the transformation of modern society, but criticized its limited social-theoretical specification of that relation. From Parsons, Luhmann inherited the attempt to theorize culture and meaning systematically, while rejecting the idea of culture as an autonomous system that determines social structure. His analysis of semantics therefore sought to reconstruct how society generates and transforms its own vocabularies, concepts, and self-descriptions in relation to changes in social structure (Luhmann, 1980: 7–17). In this sense, *Gesellschaftsstruktur und Semantik* was not a secondary historical supplement to Luhmann's general theory of society. It developed in parallel with that theory and provided a historical-sociological program for examining how changes in social differentiation became observable through semantic transformations. In *Gesellschaftsstruktur und Semantik* in particular, Luhmann drew on a wide range of materials, including historical scholarship, conceptual dictionaries, literary texts, moral treatises, political and state theory, and writings on education, in order to trace, at the level of semantic change, the transition from stratified differentiation to functional differentiation (Luhmann, 1980).

In its standard formulation, functional differentiation refers to a form of social differentiation in which relatively autonomous functional domains—such as law, politics, the economy, science, or education—reproduce themselves according to their own communicative logics and problem orientations. However, taking up Luhmann's systems theory, and especially his theory of functional differentiation, for historical-sociological and empirical research requires engaging with several lines of criticism. First, systems theory has been criticized for its high level of abstraction and for its limited capacity to grasp the complexity of interactional and practical situations (Knorr-Cetina, 1992). Questions have also been raised about the theoretical construction of differentiation at the level of society as a whole: how should “society” be specified as a unit of analysis, and on what empirical grounds can the emergence of functional systems be identified (Schwinn, 2001; Nassehi, 2004; Holzinger, 2017)? Second, these problems become even more acute in relation to world society and functional differentiation. Describing functional differentiation as the primary form of differentiation in modern society risks turning Western modernity into a normative standard and treating non-Western societies or peripheral modernities as deviations from it (Gonçalves, 2016; Holzer, 2007, 2018; Neves, 2006, 2026).

These criticisms are especially relevant when Luhmann's theory is used as a structural proposition according to which modern society is functionally differentiated. Rather than conceiving functional differentiation as a uniform structural pattern throughout world society, these approaches have emphasized its uneven realization and regionally specific configurations under different historical conditions (Neves, 2026; Albert and Stetter, 2018; Morikawa, 2018). Yet these revisions often continue to ask whether functional differentiation was achieved, blocked, delayed, or only partially realized in particular societies. In doing so, however, they risk reintroducing into the theory of functionally differentiated world society a comparative framework that treats societies as discrete, internally coherent, and mutually isolated entities—an assumption that has also shaped many theories of multiple modernities. This problem becomes especially visible in imperial and colonial contexts. Although Luhmann's theory of world society addresses the worldwide expansion of functional differentiation and the chains of inclusion and exclusion accompanying it, it pays far less attention to the historical formation of modern world society itself, particularly its entanglement with colonial rule and imperialism. Later developments of systems-theoretical world society theory have further historicized this perspective, particularly by examining the emergence of world society through processes of global interconnection and European expansion (Stichweh, 2000, 2010). Empires and colonial regimes did not operate within self-contained societies. They connected metropolitan and colonial administrations, legal orders, labor markets, police organizations, commercial actors, and local practices across territorially dispersed and hierarchically structured settings (Bhambra, 2007; Go and Lawson, 2017; Bhambra and Holmwood, 2021; Leanza and Paul, 2021).

The problem is therefore not only how unevenly functional differentiation is realized, but also whether territorially or culturally bounded societies should be treated as the primary units of analysis in the first place. Luhmann's own account, however, also provides a different point of departure. Functional systems are not simply given as institutional domains; relations between problems and their solutions are constituted through recursively connected operations. Operations refer to concrete communicative events through which distinctions become connected and reproduced. Their attribution to functionally specific contexts is stabilized through second-order observation, while guiding distinctions become empirically visible through their repeated use in vocabularies, decisions, and organizational procedures (Luhmann, 1987, 1997: 756ff., 766ff.).

Nassehi (2008) develops this operational implication more explicitly by shifting the focus from presupposed structures to the ways in which operations generate connections in the present. Functional analysis can likewise be understood not as a procedure that reduces a phenomenon to a fixed function, but as a way of asking how relations between problems and solutions are constituted within observable operations.

From this perspective, the task of historical-sociological analysis is not to determine whether functionally differentiated systems already existed as completed structures. Rather, it is to reconstruct how functionally specific distinctions were used in concrete historical situations to formulate problems, connect different modes of observation, and organize their solutions. Historical analysis therefore reconstructs how concrete problems such as labor, mobility, population, protection, or hygiene became connected through different functional distinctions and organizational procedures.

Colonial governance offers an important site for examining this task in a concentrated form. As studies of colonial governmentality have shown, colonial rule operated not only through command and direct repression, but also as a political rationality that dismantled existing forms of life and constructed new possibilities of action, subject formation, spatial order, and population registration (Scott, 1995; Kalpagam, 2000). In colonial governance, problems of labor, mobility, population, and protection were formulated and processed through functionally specific distinctions and organizational procedures. These operations were conditioned by racialized and gendered asymmetries and by the continuing possibility of police and physical coercion.

In order to examine this operational nexus, recurrent connections among heterogeneous communicative operations around a particular problem, this article focuses on the concentration and limitation of violence, that is, on the differentiation of the political system. The formation of modern society has often been described in terms of the state's monopoly of physical violence, the suppression of private violence, and the control of violence through legal and administrative procedures. In this sense, the potentialization of violence provides an important point of entry for observing the differentiation of the political system from the perspective of functional differentiation. This problem, however, cannot be contained within a history of state formation internal to Europe. In colonial governance, violence that seemed to have been backgrounded in the metropole through the formation of the constitutional state and the welfare state appeared in different forms through administrative orders, police activities, coerced labor, restrictions on mobility, hygiene policy, and population management.

This article therefore examines the potentialization of violence and its reconnection in German South West Africa by focusing on vocabularies of protection and proper treatment within an operational nexus in which multiple forms of observation intersect. German South West Africa after the Herero and Nama War is selected as a concentrated case of colonial reorganization after genocidal violence. Here, the colonial administration sought to stabilize rule by linking surviving Africans to registration, mobility control, labor contracts, and police supervision. The analysis combines colonial ordinances and selected contemporary materials with existing historical scholarship in order to reconstruct how labor and protection became observable as connected problems of colonial governance.

## Political differentiation and the potentialization of violence

Observing functional differentiation in operation requires reconsidering the place of violence in the differentiation of the political system. Modern politics has often been discussed in relation to the state's concentration of physical violence. Luhmann's theory of power as a communication medium approaches this issue not from the standpoint of the exercise of violence itself, but from the question of how the possibility of violence is maintained within political communication. Power is a symbolically generalized communication medium that increases the probability that one person's action will be connected to another's (Luhmann, 1975: 9ff.; Luhmann, 1997: 355ff.). Negative sanctions play a central role in this operation. They stabilize power situations not only when they are actually applied, but also when they are prepared, held in reserve, and backgrounded as options to be avoided by those involved.

Physical violence constitutes the extreme case of such negative sanctions. Violence functions as a symbiotic mechanism that connects the medium of power to human beings as embodied persons, thereby providing the bodily and physical possibility on which power depends as a social operation (Luhmann, 1975: 61ff.; Luhmann, 1981, 1997: 381ff.). Yet the fact that the political system holds violence as a background condition does not mean that it constantly exercises violence. Power operates not only in situations where violence is actually applied, but also when violence is anticipated as something that could be used and is therefore avoided (Luhmann, 1975: 64f.; Luhmann, 1997: 356, 380). This article refers to this relation as the potentialization of violence (Sakai, 2022). The term denotes the process through which physical violence withdraws from direct exercise and is placed in the background of political communication as a possibility of use.

The differentiation of the political system can be understood as a process that stabilizes this potentialization organizationally. The possibility of violence is placed within political operations through the state, administration, police, legal procedures, jurisdiction, and decision programs (Luhmann, 1975: 98f.; Luhmann, 1997: 382ff.; Luhmann, 2000: 55; Wansleben, 2016: 293ff.). This placement should be understood less as a simple enclosure of violence within a pre-given political domain than as an organizational mediation of the possibility of violence: who may mobilize it, through which procedures, and under what conditions. Police and administrative organizations are apparatuses capable of exercising physical coercion, but they are also interfaces that connect this possibility of coercion to forms of decision, jurisdiction, procedure, and legitimacy. What is reorganized here is therefore not only the capacity for physical coercion, but also its authorized agents, permissible limits, procedural forms, and modes of justification.

The potentialization and organizational reconfiguration of violence also involve communicative evaluations of its legitimacy. The concentration of physical coercion within political organizations raises recurring questions about who may exercise violence, for what purposes, and within what limits (Weber, 1919). Particular forms of coercion are thereby presented, contested, and evaluated as legitimate or illegitimate within concrete interactions and organizational settings. Moral semantics provide resources through which such evaluations are articulated, stabilized, criticized, and revised. In Luhmannian terms, moral communication operates across different functional contexts by communicating conditions of respect and disrespect (Luhmann, 1997: 396ff.; Luhmann, 2008). Political decisions therefore become one arena in which moral distinctions are mobilized, contested, and temporarily stabilized without converging on a single authoritative standard.

This understanding allows us to describe the differentiation of the political system as an operational nexus that involves both the concentration and the limitation of violence. The modern state holds physical violence as a background condition of the medium of power, while connecting its exercise to procedures of organization such as administration and police. This perspective is important for understanding the transition from the *Polizeistaat* to the constitutional state, as well as the process through which comprehensive police concern was reorganized into administrative, legal, and welfare-state institutions. At the same time, if this process is treated as a history of state formation internal to Europe, it becomes difficult to see how the possibility of violence was rearranged within imperial and colonial relations. The next section therefore examines how potentialized violence and manifest physical coercion were reconnected within multiple problem formulations in colonial governance.

## Colonial violence and the operational reconfiguration of power

Studies of violence in colonial governance have raised important questions for theories of the state, power, and violence that have taken Western modernity as their point of departure. Theories of modern state formation have treated the concentration of dispersed violence, the suppression of private violence, and the control of violence through legal and administrative procedures as central moments in state formation (Weber, 1919; Elias, 1939; Tilly, 1985). Within this framework, violence is concentrated in the state, while its exercise is limited through law and administration and pushed into the background of everyday social relations. Colonial studies have challenged this understanding by showing that the concentration of violence does not necessarily lead to its restraint. In colonial governance, violence was incorporated into the ordinary operations of rule through land dispossession, restrictions on mobility, forced labor, population classification, legal status differentiation, and the formation of settler society.

As Wolfe (2006) famously argued, settler colonialism should be understood not as an event but as a structure. From this perspective, colonial violence is not confined to moments of conquest or war. It continuously links the elimination of Indigenous societies to the construction of settler society. Veracini (2010) similarly describes settler colonialism not only as a system of domination or exploitation, but also as a political project aimed at founding another society. Violence is thereby woven into social formation itself through land acquisition, migration, segregation, assimilation, and elimination.

Theories of the colonial state and studies of colonial governmentality have also examined violence in relation to sovereignty, states of exception, legal classification, and administrative status. Mbembe's (2003) account of necropolitics understands the colony as a privileged site in which sovereignty operates as the power to expose people to death. Mamdani's (1996) analysis of the colonial state shows how violence under colonial rule was institutionalized through the distinction between native and citizen, customary law, indirect rule, and administrative classification. Scott's (1995) account of colonial governmentality likewise understands colonial governance as a political rationality that dismantles existing forms of life and constructs new possibilities of action, subject formation, and spatial order. Taken together, these arguments show that violence in colonial settings was not concentrated only in moments of war or conquest. It was continuously organized through law, administration, population registration, labor control, and spatial ordering. Colonial violence was also organized through normative distinctions that evaluated particular forms of coercion as legitimate or illegitimate, acceptable or excessive. Such evaluations helped define the conditions under which violence could be justified, limited, or criticized within colonial rule.

Against the background of these critiques, Luhmann's theory of the political system may also be read as a theory of violence that presupposes a history of state formation internal to Europe. At first glance, the idea that physical violence is potentialized in the background of the medium of power, and that its exercise is controlled through the state, administration, legal procedures, and police, seems to share the same problem as the Western-modern model of state formation criticized by colonial studies. The question, then, is whether an understanding of violence as concentrated within the political system, proceduralized, and backgrounded can adequately grasp the visibility, dispersion, and everyday character of violence in colonial governance.

As the previous section suggested, however, Luhmann's systems theory can be read operationally. From this perspective, the potentialization of violence is not simply a concept for describing a completed model of the modern state. It offers a way of asking how the possibility of physical coercion is held in reserve, limited, and connected through concrete procedures and organizations. The colonial case shifts the question accordingly. What matters is how physical coercion, made visible in war and coercive labor, was reconnected to the ordinary operations of rule. The colonial case also raises the question of how racialized and gendered relations shaped the moral evaluation of who may exercise violence, against whom, and within what limits.

In colonial governance, physical coercion was reconnected to ordinary rule through police action, administrative decisions, labor contracts, and pass regulations. Vocabularies of protection and proper treatment provided resources for evaluating these arrangements and for distinguishing permissible coercion from abuse or excess. The application of these distinctions was shaped by racialized and gendered asymmetries, which distributed the capacity to exercise violence and claims to protection unevenly between colonizers and colonized subjects. This operational nexus provides the point of entry for the present article's attempt to develop the operational implications of Luhmann's systems theory. From this perspective, the differentiation of the modern political system involves the potentialization of physical violence as a background condition of the medium of power and its organizational mediation through decisions, procedures, and jurisdictions. Under colonial conditions, this latent possibility was repeatedly activated and reorganized through the governance of labor, worker treatment, and protection, while police enforcement and settler punishment brought physical coercion back into view. A functionalist empirical analysis therefore traces how relations between problems and solutions were constituted in concrete operations and how normative distinctions shaped the limits and legitimacy of coercion. The next section turns to German South West Africa after the Herero and Nama War and examines how labor contracts, proper treatment, and protection connected labor governance to the continuing possibility of physical coercion.

## Colonial labor governance, police mediation, and protective treatment

### War, camps, and the making of labor power

German South West Africa (Deutsche Südwestafrika, DSWA) became a German “protectorate” (*Schutzgebiet*) in 1884, but it did not initially form an integrated colonial state with effective control over the territory. Early DSWA was an unstable space of rule in which different claims overlapped: the international declaration of protectorate status, concessions granted to private companies and merchants, protection treaties with local chiefs, imperial claims to superior authority and to land and mineral resources, and the rights of use and residence held by missionaries (Bley, 1968: 66ff.; Conrad, 2008: 29; Gründer, 2022: 85ff.). Over time, however, DSWA developed as the German Empire's only large-scale settler colony. Its trajectory increasingly aimed at reorganizing land use, population placement, and labor relations according to the interests of settler society and the colonial economy (Conrad, 2008: 29f.; Leanza, 2020; Zimmerer, 2004: 3). What was at stake in this settler-colonial project was not only the extraction of African labor, but also the separation of Indigenous populations from land and their relocation as labor power available to the colonial economy (Muschalek, 2019: 130).

The Herero and Nama War, which began in 1904, violently accelerated this colonial reorganization. The Herero uprising emerged from a cumulative crisis: the gradual loss of territory, the weakening of traditional authority, restrictions on land use, settler violence and property violations, indebtedness to traders and farmers, and the destruction of the pastoral economy by the rinderpest of 1896–1897 (Bley, 1968: 133, 157f.; Zimmerer, 2004: 26f., 31; Gründer, 2022: 127). For the Herero, cattle sustained wealth, status, relations of protection, mobility, and political authority. Their loss undermined the conditions of Herero autonomy and pushed many into dependence on settler farms and wage labor. The German campaign culminated in the Battle of Waterberg, after which Herero communities were driven into the Omaheke. From late 1904 onward, the conflict expanded through the Nama resistance across the southern regions of the colony and continued until 1908 (Bley, 1968; Gründer, 2022). Taken together, these campaigns created the conditions for the postwar reorganization of colonial rule.

The military campaigns did not end with the defeat of armed resistance. After Waterberg, German forces sealed off waterholes in the Omaheke and pursued not only combatants but also families, causing large-scale mortality among the Herero (Bley, 1968: 191, 202). At the same time, survivors from both the Herero and Nama campaigns were concentrated in camps such as Swakopmund and Shark Island, where overcrowding, malnutrition, disease, guard violence, and forced labor produced devastating demographic consequences (Zeller, 2004: 64; Erichsen, 2004: 80ff.; Kreienbaum, 2015: 121ff.). These camps were not merely remnants of wartime exterminatory policies. They also anticipated the postwar colonial order by concentrating, classifying, and reallocating surviving Africans as labor power.

**Figure 1:**
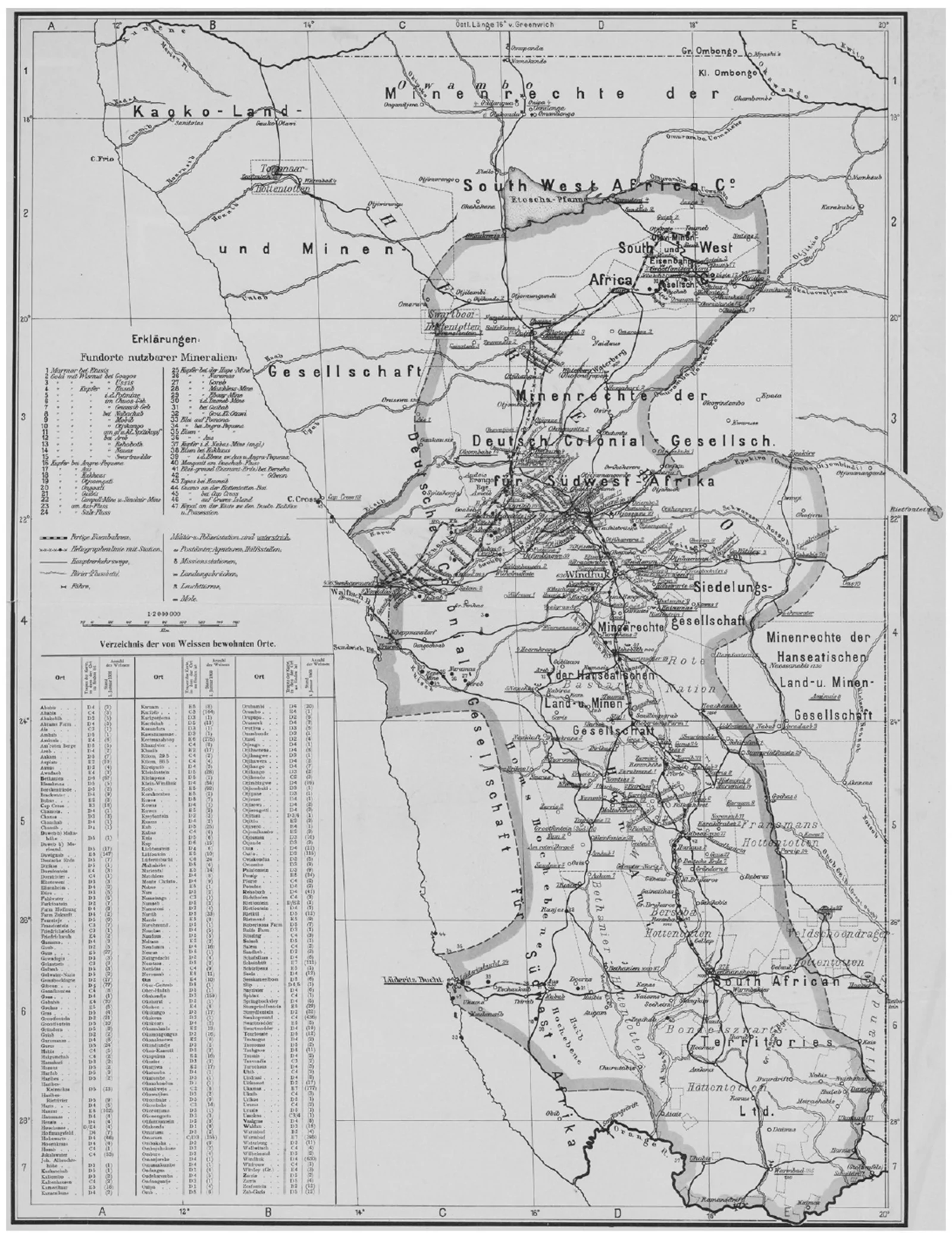
German South West Africa and the area designated for governmental police protection under the 1907 Native Ordinances. Source: Preußischer Kulturbesitz (1907).

In this respect, the physical coercion made manifest in the Herero and Nama War formed an institutional precondition for the new colonial order. The so-called Native Ordinances of 1907 (*Eingeborenenverordnungen*) belonged to this transformation (see Figure 1). They institutionalized the destruction produced by war and camps through a legal and administrative framework through which surviving Africans could be made available to the colonial economy (Zimmerer, 2004: 56, 77ff.; Eingeborenenverordnungen, 1907: Nr. 212–214).

### Registration, passes, and racialized population observation

The order that emerged through war and camps could not be maintained by labor mobilization alone. If Africans were to be used as labor power, colonial administration had to identify individuals, trace their movements, and attach them to employment relations. The Native Ordinances of 1907 connected registration, passes, residence control, and labor contracts for this purpose. In this arrangement, the colonized were observed as subjects whose movements could be controlled, whose labor could be allocated, and whose lives could be placed under police supervision.

The Native Ordinances of 1907 consisted of three regulations: the control ordinance (*Verordnung betreffend Maßregeln zur Kontrolle der Eingeborenen*), the pass ordinance (*Verordnung betreffend die Paßpflicht der Eingeborenen*), and the servant and labor contract ordinance (*Verordnung betreffend Dienst- und Arbeitsverträge mit Eingeborenen*). The control ordinance made registration in the native register the administrative basis of rule. It also placed land rights and the possession of large livestock under a permit system and introduced provisions for punishing vagrancy. The pass ordinance regulated mobility through the pass mark (*Paßmarke*) and travel pass (*Reisepaß*) and prohibited the employment, lodging, maintenance, or support of persons without valid passes. The servant and labor contract ordinance connected labor contracts to police confirmation through the service book (*Dienstbuch*) (Eingeborenenverordnungen, 1907: Nr. 212–214; Zimmerer, 2004: 56, 77ff.). Taken together, these ordinances linked registration, mobility, and employment as mutually reinforcing techniques of governance.

The distinctive feature of this institutional arrangement was that registration and mobility control were closely tied to the observation of labor power. The pass mark had to be shown not only to police authorities but also to white residents, thereby connecting state control to the everyday surveillance exercised within settler society (Eingeborenenverordnungen, 1907: Nr. 212, §§3–4; Nr. 213, §§2–3, 14–16; Nr. 214, §§1–2). The pass system thus made the colonized visible to the colonial state while also making them identifiable and manageable in everyday encounters with white residents.

The form of population observation at work here did not treat colonizers and colonized people as members of a single comparable population. As Renard (2021) shows with regard to German colonial statistics, colonial population registration placed colonizers and colonized people under different principles of classification. The latter were observed primarily through questions of labor, mobility, hygiene, security, and governability. From this perspective, registration, passes, and service books in the Native Ordinances of 1907 were institutional techniques for making the colonized observable in a specific way.

Registration, passes, and service books did not exercise physical coercion directly. They inserted the possibility of such coercion into everyday administrative forms. Movement without a valid pass, departure from an employment relation, unregistered residence, or unauthorized support could all become matters for police intervention. The colonized were thereby constituted as persons who could be checked, stopped, returned, and reallocated. Through these devices, the labor power formed through war and camps was connected to the semi-free labor market.

### Labor contracts and the police mediation of semi-free labor

Africans who had been grasped as individuals through registration, passes, and service books were connected to the semi-free labor market through the institutional form of the labor contract. The labor order established after the Native Ordinances of 1907 treated Africans as workers who could enter into employment contracts. The servant and labor contract ordinance made the issuing of a service book (*Dienstbuch*) a condition for the validity of service and labor contracts lasting longer than one month. It also required police authorities to confirm whether a prior contract existed, whether the worker understood the terms of the contract, and whether consent had been given (Eingeborenenverordnungen, 1907: Nr. 214, §1). Within this form, African workers were understood not only as objects of direct coercive mobilization, but also as parties to contractual relations.

This contractual form operated on the basis of registration, mobility control, and police confirmation. Zimmerer describes this arrangement as a “semi-free” labor market because the vocabulary of contract, choice, and consent was embedded in a wider system that combined dispossession, mobility control, and administrative supervision of labor supply (Zimmerer, 2004: 176ff.). The colonial government faced this problem under conditions of severe labor scarcity. Agriculture, mining, railway construction, and harbor works continued to depend on cheap African labor, while the war had dramatically reduced the available labor force. Securing workers, preventing desertion, and tying them to employment relations thus became central tasks of colonial governance.

The *Landespolizei* mediated this labor market in everyday practice. In DSWA after the Herero and Nama War, the police linked the registration of workers to the supervision of contracts and the pursuit of deserters. As Muschalek shows, it secured workers, allocated them, handed them over, and redistributed them (Muschalek, 2019: 140–141). Police searches also drew settlers and colonized intermediaries into the colonial project and made it more difficult for Africans to withdraw from the labor regime (Muschalek, 2019: 140).

Labor contracts therefore did not remain a bilateral relation between employer and worker. They were embedded in a wider police-mediated arrangement that linked employment to registration, mobility control, and the pursuit of deserters. Workers entered employment relations through contracts, but departure from those relations or unauthorized movement could immediately become a matter for police handling. In this sense, the contract recorded the availability of labor, monitored detachment from employment, and made such detachment administratively actionable.

The semi-free labor market was thus an operational nexus in which contractual forms and police mediation reinforced one another. Here, the possibility of physical coercion was incorporated into the maintenance of employment relations, the control of movement, and the handling of desertion. The next question is how desertion, disobedience, and employer punishment were processed within this labor market, and how these practices became connected to the vocabularies of proper treatment and *Fürsorge*.

### Punishment, proper treatment, and police-mediated labor governance

Within the semi-free labor market, the possibility of physical coercion became problematic in two related ways. The first concerned the handling of desertion, refusal, disobedience, breach of contract, and employers' suspicions. Here the question was how much physical coercion could be permitted in order to keep African contract workers under the supervision and disciplinary authority of employers. The second concerned the maintenance of workers as usable labor power. In this context, punishment, protection, and proper treatment became connected as conditions for preventing desertion and preserving labor capacity.

The physical coercion at stake in the first dimension appeared in the problem of “gross abuse” (*grobe Mißhandlung*) under Article 7 of the 1907 servant and labor contract ordinance. This article stipulated that Indigenous workers could terminate a service contract during its term on the grounds of gross abuse or serious violation of the employer's obligations (Eingeborenenverordnungen, 1907: Nr. 214, §7; Zimmerer, 2004: 199). In January 1908, the colonial government in Windhoek, through a circular issued by Governor Seitz, asked district offices to report on how “gross abuse” against Indigenous workers should be assessed. The responses show that the boundary between abuse (*Mißhandlung*) and punishment (*Züchtigung*) was a matter of practical adjustment. In some districts, beatings with whips or sticks that left workers unable to walk were treated as abuse. In other cases, beatings for refusal to work or violation of duties could be handled as “light corporal punishment” (*leichte körperliche Züchtigung*) rather than gross abuse (Zimmerer, 2004: 201ff.). Physical coercion was thus placed at the boundary between permissible punishment and punishable abuse.

The legal and moral reclassification of colonial violence became particularly visible when violence itself became the object of judicial proceedings. Between 1911 and 1913, several criminal trials concerned severe abuse committed by white farmers against African workers (Bley, 1968). Among them, the criminal proceedings against the farmer Ludwig Cramer illustrate how the same acts of physical coercion were reconstructed through different legal and semantic distinctions. Following the disappearance of livestock and suspicions of cattle poisoning, Cramer interrogated African workers, beat them with a sjambok, and detained them in order to obtain statements. His defense presented these acts as legitimate disciplinary measures intended to compel the workers to tell the “truth” (Bley, 1968: 299).

The proceedings reformulated Cramer's descriptions of his conduct through different legal categories. His claim that African workers owed him *Rechenschaftspflicht* (a duty of accountability) became the legal question of *Erpressung eines Geständnisses* (coercion to obtain a confession). His description of cattle poisoning as *ein Angriff in Permanenz* (a continuing attack) was translated into the question of whether a *gegenwärtiger Angriff* (present attack) existed for the purposes of self-defense. His appeal to a *väterliches Züchtigungsrecht* (paternal right of punishment) became the question of whether disciplinary authority over African workers could legitimately be derived from §1631(2) of the German Civil Code, §127 of the Trade Regulation Act, and older servant ordinances (Der Südwestbote, 1912a,b; Bley, 1968: 299–300). Through witness examination, legal qualification, and judicial reasoning, the same acts of physical coercion were thereby translated into distinct legal and administrative problems. The surviving court records document this process of legal reconstruction in the organization of testimony, evidence, charges, and judicial decisions. Even after the introduction of labor contracts, a racialized and paternalistic *Herr- und Diener-Verhältnis* continued to provide the framework through which punishment against African workers was interpreted and justified (Renard, 2025: 241f.).

The second dimension concerned “proper treatment” as a condition for maintaining labor power. In his circular of January 1908, Seitz stated that only “calm, even, and just treatment” (*ruhige, gleichmäßige und gerechte Behandlung*) could turn Indigenous people into “willing and satisfied workers.” He also argued that workers would be less inclined to flee their masters if they were convinced that they were not mere slaves, that their legitimate claims were being met, and that they stood under the “protection of the law” (Zimmerer, 2004: 199). Treatment here concerned the material conditions of labor maintenance, including food, housing, wages, clothing, and care in cases of illness. Article 7 of the servant and labor contract ordinance defined gross abuse and serious violations of employers' duties as grounds for terminating a contract, but it also required employers to provide maintenance, bandages, and medicines during the first 4 weeks of a worker's illness (Bley, 1968: 212; Eingeborenenverordnungen, 1907: Nr. 214, §7). District officials likewise evaluated service books and written contracts as instruments that could prevent desertion while also protecting workers from abuse and exploitation (Zimmerer, 2004: 84ff.).

The conditions for keeping workers within employment relations were further specified in a report by Vietsch, the district officer in Rehoboth. Vietsch understood desertion not primarily as a problem of insufficient punishment or of workers' character, but as a problem of inadequate food and payment. He therefore proposed prohibiting the truck system and standardizing food provision (Muschalek, 2019: 152f.). A similar logic appears in a December 1908 document sent by Police Inspector Hildebrandt of the Waterberg police depot to the farmer Freiherr von Speth. Hildebrandt stated that the supply of workers depended on police care and on employers' proper treatment of African workers. Employers who maintained workers through adequate food and good treatment could expect police support if workers became lazy or insolent; those who failed to meet these conditions could not expect such assistance (Muschalek, 2019: 144). Proper treatment thus functioned as a condition under which employers could claim police support.

Dernburg's call for “heightened care” (*erhöhte Fürsorge*) in Windhoek in 1908 can be situated in the same context. As head of the Imperial Colonial Office in Berlin, Dernburg justified this need by pointing to the sharp decline of the African population after the “uprising and campaign.” In his view, the interests of the colony itself required greater care. What he had in mind was the maintenance of Africans as present labor power and as a future healthy population through sanitary protection, food provision, milk for the Herero in particular, and the possibility of acquiring large livestock (Bley, 1968: 267). In this context, *Fürsorge* did not remove the possibility of punishment. It connected labor maintenance, employers' treatment of workers, police support, and population preservation.

Punishment, proper treatment, and *Fürsorge* were thus connected within the same police-mediated labor governance: workers were to be kept within employment relations, maintained as usable labor power, and made available for intervention when necessary. This nexus operated within a racialized and gendered order. African workers were treated as parties to labor contracts through service books and contractual forms, while also being observed as subjects to be managed, protected, and punished through pass control, registration, and paternalistic employer authority. Although the semi-free labor market primarily targeted mobile African male workers, police-mediated labor governance also reached into the reproduction of labor power through family regulation, women's auxiliary and unpaid labor, and the socialization of young men (Muschalek, 2019: 32–35, 99, 101). In this way, vocabularies of protection and proper treatment were formed within a labor regime that connected maintenance, reproduction, and punishability.

## Conclusion: functional distinctions and colonial observability

This article has examined how colonial violence was reorganized in German South West Africa after the Herero and Nama War through a regime of registration and labor control. Physical coercion made manifest through war and camps was connected to procedures that formed labor power, supervised employment relations, enabled police intervention, and maintained workers as usable labor. Violence was therefore not concentrated only in moments of conquest or genocide. It was incorporated into the everyday operations of colonial governance as a condition for constituting laboring subjects who could be registered, employed, disciplined, and maintained.

What this case shows for the thesis of the potentialization of violence is that the backgrounding of physical violence and its manifestation as bodily coercion were closely intertwined in colonial governance. In Luhmann's theory of power, physical violence is held as a possibility at the extreme point of negative sanctions. In German South West Africa after the Herero and Nama War, this possibility was distributed within labor governance through administrative records, police practices, employers' disciplinary authority, and vocabularies of protection and proper treatment. The central question is therefore not whether violence was latent or manifest. It is how the possibility of physical coercion constituted, maintained, and made governable the objects of colonial rule through specific forms of observation.

This has methodological implications for taking up the theory of functional differentiation operationally. In the case of DSWA, legal, contractual, protective, and police vocabularies became relevant through the problems they made visible and the forms of processing they enabled. Labor contracts established employment relations, but through service books and police confirmation they also made workers' movement and detachment from employment administratively actionable. Vocabularies of protection and proper treatment problematized workers' living conditions while connecting them to the prevention of desertion, police support for employers, and the maintenance of labor power. Functional distinctions thus operated as forms of observation that constituted relations between problems and their processing within concrete operations.

This operational nexus acquired its objects through racialized and gendered asymmetries. African workers were treated as parties to labor contracts through service books and contractual forms, while at the same time they were observed as subjects to be managed, protected, and punished through pass control, registration, master-servant relations, and police supervision. The maintenance of labor power also depended on gendered arrangements that included women's auxiliary and unpaid labor, the socialization of young men, and the regulation of family life. In this way, vocabularies of contract, legal protection, proper treatment, and *Fürsorge* operated as forms of observation that connected the possibility of physical coercion to labor governance under racialized and gendered conditions.

This article has argued that a Luhmannian approach to functional differentiation can be developed as an operationally grounded historical sociology. The central task is to examine how concrete practices of rule made certain problems observable as legal, economic, policing, protective, or educational matters. In DSWA after the Herero and Nama War, violence, labor, law, policing, and protection were connected through racialized and gendered forms of observation. This offers one historical-sociological point of departure for rethinking the potentialization of violence within the global formation of modernity.

## Data Availability

The original contributions presented in the study are included in the article/supplementary material, further inquiries can be directed to the corresponding author.
